# An Integrated Metabolomics Study of Glucosinolate Metabolism in Different Brassicaceae Genera

**DOI:** 10.3390/metabo10080313

**Published:** 2020-07-31

**Authors:** Yu Liu, Merja Rossi, Xu Liang, Hui Zhang, Li Zou, Choon Nam Ong

**Affiliations:** 1 State Key Laboratory of Reproductive Regulation and Breeding of Grassland Livestock, School of Life Sciences, Inner Mongolia University, Hohhot 010070, China; yuliu@imu.edu.cn; 2 Saw Swee Hock School of Public Health, National University of Singapore, Singapore 117549, Singapore; merja.rossi@gmail.com (M.R.); ephzouli@nus.edu.sg (L.Z.); 3 National University of Singapore (NUS) Environmental Research Institute, National University of Singapore, Singapore 117411, Singapore; liangxu.1210@gmail.com (X.L.); zhanghui@u.nus.edu (H.Z.)

**Keywords:** Brassicaceae, metabolic profiling, pathways, glucosinolates, isothiocyanates, amino acids

## Abstract

Glucosinolates are a group of plant secondary metabolites that can be hydrolyzed into a variety of breakdown products such as isothiocyanates, thiocyanates, and nitriles. These breakdown products can facilitate plant defense and function as attractants to natural enemies of insect pests. As part of the diet, some of these compounds have shown cancer-preventing activities, and the levels of these metabolites in the edible parts of the plants are of interest. In this study, we systematically examined variations in glucosinolates, their precursors, and their breakdown products in 12 commonly consumed vegetables of the Brassicaceae family with gas chromatography—quadrupole time-of-flight mass spectrometer (GC-Q-TOF/MS), liquid chromatography–quadrupole time-of-flight mass spectrometer (LC-Q-TOF/MS), and liquid chromatography—triple quadrupole mass spectrometer (LC-QQQ/MS), using both untargeted and targeted approaches. The findings were integrated with data from literature to provide a comprehensive map of pathways for biosynthesis of glucosinolates and isothiocyanates. The levels of precursor glucosinolates are found to correlate well with their downstream breakdown products. Further, the types and abundances of glucosinolates among different genera are significantly different, and these data allow the classification of plants based on morphological taxonomy. Further validation on three genera, which are grown underground, in damp soil, and above ground, suggests that each genus has its specific biosynthetic pathways and that there are variations in some common glucosinolate biosynthesis pathways. Our methods and results provide a good starting point for further investigations into specific aspects of glucosinolate metabolism in the Brassica vegetables.

## 1. Introduction

Glucosinolates are unique and prevalent secondary metabolites found in the order Brassicales [1]. This order includes the economically important family Brassicaceae, consisting of many common vegetables such as broccoli, cabbage, Chinese cabbage, radishes, watercress, and rocket [1]. Glucosinolates act as precursors for compounds with anti-carcinogenic properties [2]. Epidemiological studies show that a diet rich in broccoli and other cruciferous vegetables reduces the risk of cancer [3,4,5,6,7].

In plants, glucosinolate breakdown pathways are known to have protective roles in both abiotic and biotic stress. During cell breakdown, endogenous β-glucosidases, also called myrosinase enzymes, get mixed with the glucosinolates in the cell, hydrolyzing the thioglucosidic bond [8]. The reaction can form isothiocyanates, which can be converted into nitriles, epithionitriles, or organic thiocyanates. Most of these compounds can evaporate and enter intact cells because most of them are volatile and lipophilic [9]. Many Brassicaceae plants have been found to have differences in the composition and content of glucosinolate metabolites [10].

The exact role of these glucosinolate metabolites remains unknown. However, isothiocyanates specifically have been shown to be toxic to many organisms that may be harmful to plants, such as insects, microorganisms, and nematodes [11,12,13]. In aboveground plants, the roots are known to contain higher concentrations of a larger variety of glucosinolates than shoots [14]. The differences between different parts of the plant are, at least partly, due to the effectiveness of certain types of breakdown products in the soil. Constant high pathogen pressure on roots has also been suggested as a factor that could explain why glucosinolate levels in roots are reported to be more stable than in shoots [14].

Recent studies suggest that glucosinolates may have certain roles within undamaged cells, which could be linked to signaling and defense against pathogens [9,15]. Despite the complexity caused by genome duplication, investigations have started to shed light on the genetic variation and gene function in glucosinolate metabolism [16,17,18,19]. Combining this information with metabolomics could prove to be important in understanding the regulation of the biosynthetic pathways and complex roles of glucosinolates and their breakdown products in plants.

Understanding of glucosinolate metabolism can lead to significant improvements in breeding vegetables that have beneficial qualities [10]. An example of using traditional breeding to achieve this is the Beneforté broccoli, where segments of the genome were introgressed from a wild variety of Brassica villosa [20]. This led to 2.5–3 times glucoraphanin content due to the enhancement of sulfate assimilation and modifications in partitioning among sulfur-containing metabolites. In the future, systematic approaches in breeding that combine understanding of the metabolic pathways, availability of precursors, and the regulation of biosynthesis will benefit from metabolomics studies that generated large datasets across the complex network of glucosinolate metabolism.

A large number of studies have been conducted on glucosinolates in Brassicaceae vegetables with the aim of ultimately introducing more beneficial compounds into the diet. However, differences in extraction and analysis methods as well as experimental protocols can lead to data incomparability among different experiments [21]. Some studies have also shown that growth, harvesting, and storage conditions can affect the metabolite content [22,23,24,25,26]. More comprehensive information is therefore needed across different Brassica vegetables when considering consumption and diet.

Using an integrated approach with liquid chromatography–quadrupole time-of-flight mass spectrometer (LC-Q-TOF/MS), gas chromatography—quadrupole time-of-flight mass spectrometer (GC-Q-TOF-MS), and liquid chromatography—triple quadrupole mass spectrometer (LC-QQQ-MS), using both untargeted and targeted analysis, we quantified amino acids, glucosinolates, and isothiocyanates in the edible parts of plants grown for consumption. In addition, we summarized the currently known pathways involved in glucosinolate metabolism in Brassicaceae based on 12 commonly encountered Asian vegetables. Further, we compared the metabolic profiles of three Brassica grown under different circumstances, including radish that grows underground, watercress that grows in damp soil, and broccoli that grows above ground. The data suggest that each genus has its specific biosynthetic pathways distinguished from the others. However, there are differences in some shared glucosinolate biosynthesis pathways, such as variations in metabolite abundances. Given that the integrated approach used in this study allows comprehensive measurements of a wide varieties of relevant metabolites, the totality of data obtained here could serve as a database that is valuable for further investigations of specific aspects of glucosinolate metabolism in the Brassicaceae family.

## 2. Results

### 2.1. Profiles and Correlations among Glucosinolates and Their Precursors and Breakdown Products

First, untargeted profiling of the non-volatile and volatile metabolites of the 12 Brassicaceae vegetables was performed. After detection frequency (>100%) and low relative standard deviation (RSD < 30%) filtration, the remaining metabolites were identified, with their relative abundances presented as a heat map in Supplementary Materials Figure S1. In total, 223 metabolites were detected and identified, including 32 glucosinolates. Seventeen of these glucosinolates were then quantified with available commercial standards. Those detected glucosinolates for which standards were not commercially available [21] were thus quantified using a semi-quantitative approach. These untargeted analyses, as well as the quantitative/semi-quantitative analyses of the glucosinolates in the 12 Brassicaceae vegetables (3 batches each), were performed as a discovery data set. The glucosinolate data obtained here show profiles and trends similar to various previous studies (Supplementary Materials Figure S1). For example, rocket salad was found to be high in glucoraphanin, which confirmed the previous report of up to 52% of its total glucosinolate content [27]. Similarly, dehydroerucin and its hydrolysis product 4-methylthio-3-butenyl isothiocyanate were previously found in high abundances in radish and cherry radish [10]. Glucoerucin was detected in cabbage, cauliflower, rocket, and radishes, but not in the other Brassica genus plants in the present study. Interestingly, glucoerucin was reported to form part of the profile that acts as an attractant for the diamondback moth in some cabbage accessions [12]. These data demonstrate that different types of brassica, albeit from the same family, have their unique content of glucosinolates and volatile secondary isothiocyanate metabolites. However, it must also be considered that the various Brassica samples were obtained commercially, and thus the observed variations in glucosinolates and isothiocyanates may also be associated with differences in growth location and growth practices, as well as storage conditions and duration. Although it may be considered that vast changes in concentration and qualitative differences are likely most impacted upon by Brassica variety within this study, only by performing controlled field experiments where the different Brassica varieties are produced, harvested, and stored within identical conditions can all differences observed in glucosinolate and isothiocyanate profiles be attributed to variety alone. Furthermore, Pearson correlation analysis revealed close correlations between some specific glucosinolates and their breakdown product isothiocyanates (Supplementary Materials Figure S2). The allyl ITC, 3-Butenyl ITC, 3-Methylthionpropyl ITC, 4-Methylpentyl ITC, and Phenethyl-ITC were found to be positively correlated with their respective parental glucosinolates across the 12 vegetables. On the other hand, there was no close correlation between amino acids at the beginning of the biosynthetic pathway and the secondary metabolites glucosinolates (Supplementary Materials Figure S1). For example, we would expect methionine to be crucial for the synthesis of some of the glucosinoltes such as sinigrin, glucoiberin, glucosativin, glucoerucin, and glucoraphanin in the radish, however, the data shows very different trends between methionine and these downstream glucosinolates (Supplementary Materials Figure S1). In contrast, while watercress contains high abundance of methionine (about 150 µg/g dry weight, Supplementary Materials Table S1), the glucosinolate metabolites such as sinigrin, glucoiberin, glucoerucin, and gluconapin that are involved in the rest of the pathway were not detected at high abundance. Similar results were obtained from the quantitative analysis of amino acids and glucosinolates (Supplementary Materials Table S1).

**Figure 1:**
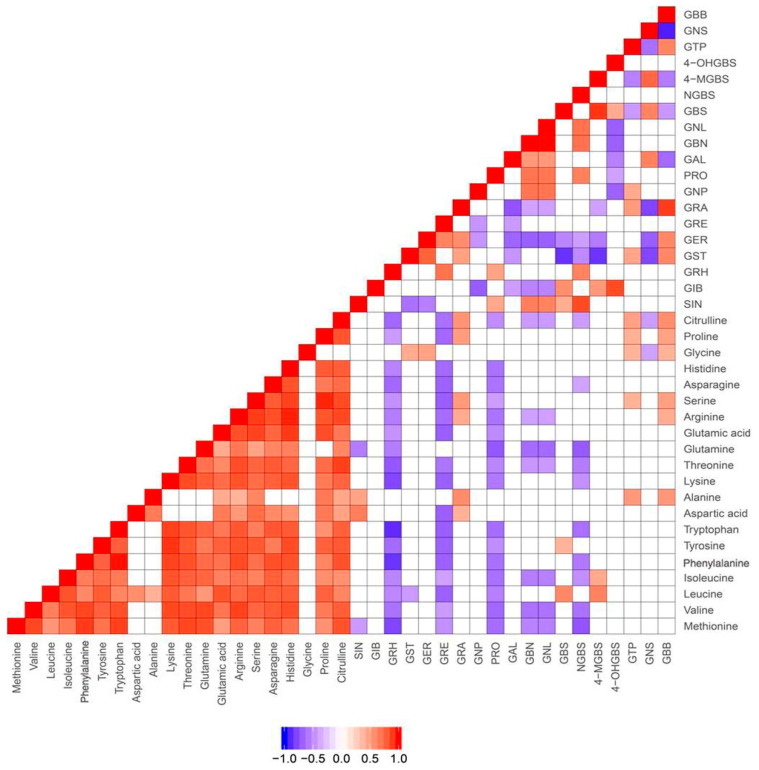
Correlations between metabolites in 5 vegetables confirmed by targeted quantitative analysis of amino acids, glucosinolates. SIN, sinigrin; GIB, glucoiberin; GRH, glucoraphasatin; GST, Glucosativin; GER, glucoerucin; GRE, glucoraphenin; GRA, glucoraphanin; GNP, gluconapin; PRO, progoitrin; GAL, glucoalyssin; GBN, glucobrassicanapin; GNL, gluconapoleiferin; GBS, glucobrassicin; NGBS, neoglucobrassicin; 4-MGBS, 4-methoxyglucobrassicin; 4-OHGBS, 4-hydroxyglucobrassicin; GTP, glucotropaeolin; GNS, gluconasturtiin; GBB, glucobarbarin.

In order to validate the above findings and further investigate the correlations between glucosinolates and their amino acid precursors, we performed targeted analysis for five of the 12 vegetables (5 batches for each vegetable). These five vegetables were broccoli, choy sum, radish, watercress, and rocket salad, which belong to the subfamilies of Brassicaceae—*Brassica oleracea, Brassica rapa*, *Raphanus*, *Nasturitum*, and *Eruca,* respectively. The quantitative results are shown in Supplementary Materials Table S1. Consistently, significant correlation was not observed between amino acids and glucosinolates (Figure 1). This might be due to the fact that amino acids can be converted to intermediates such as aldoximes and thiohydroximates, which can be subsequently converted to a number of plant secondary metabolites, including precursors for glucosinolates [28]. However, close correlations were observed among the amino acids except for glycine (Figure 1).

### 2.2. Glucosinolate Metabolites are Potentially Useful as Chemotaxonomic Markers

**Figure 2:**
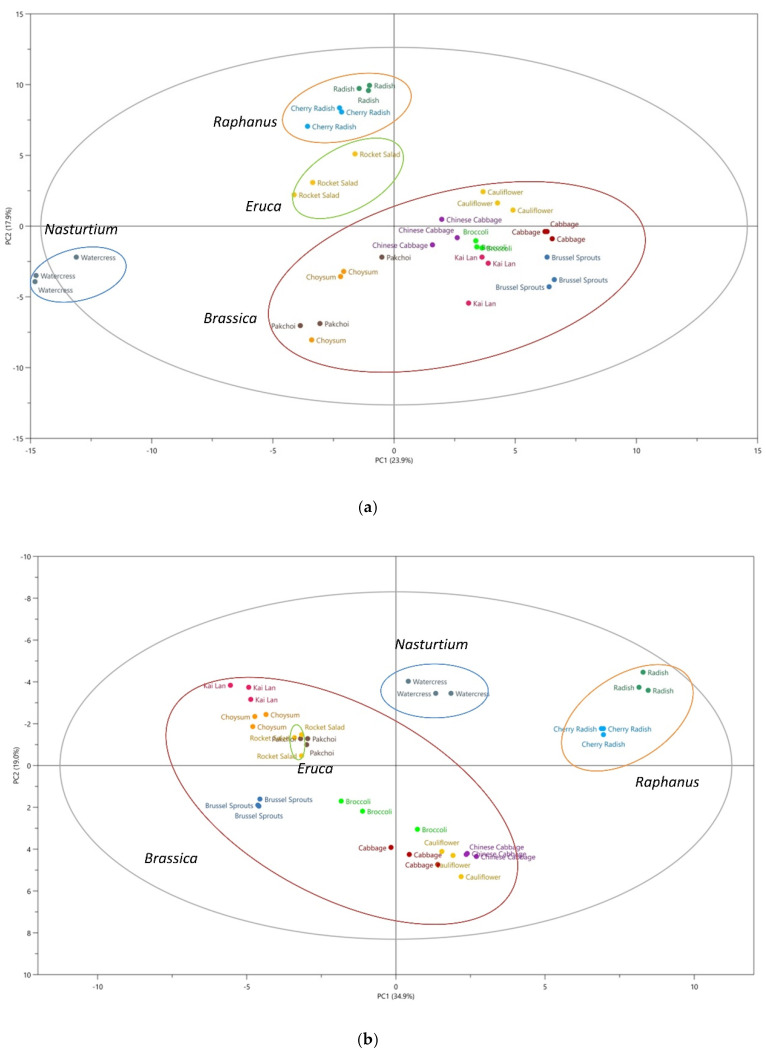

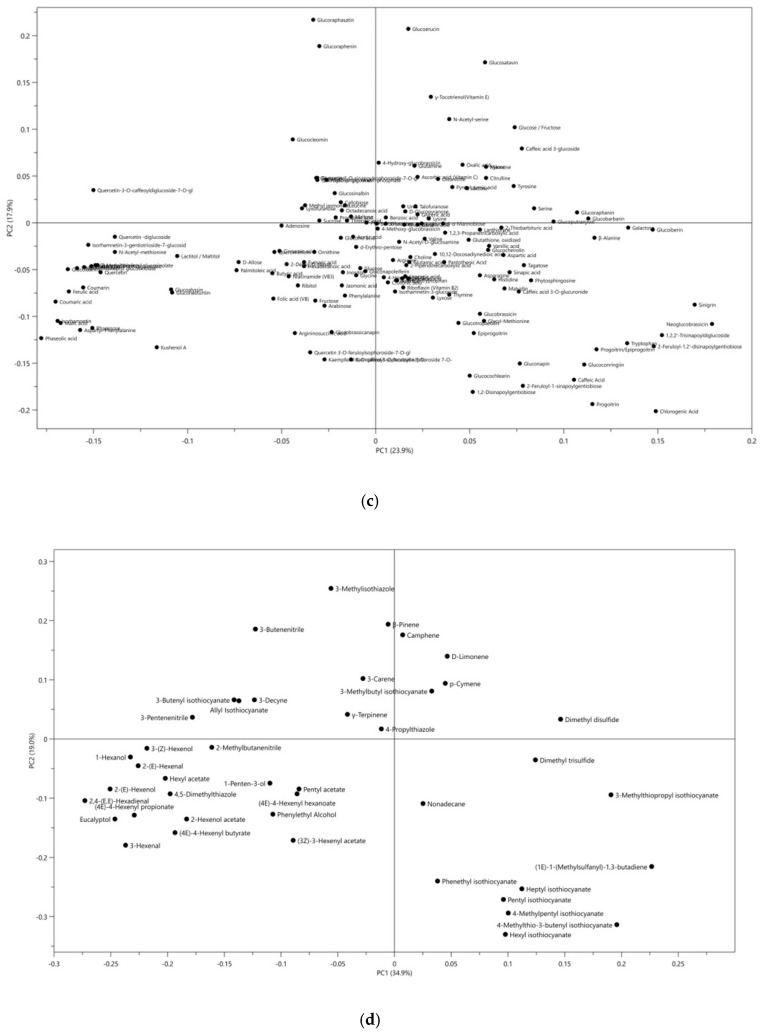
Principal component analysis (PCA) showing separation of 12 vegetables for data from two different experiments and two different datasets. (**a**) Score plot of PCA analysis based on non-volatile compounds, ●Cherry Radish, ●Radish, ●Rocket Salad, ●Watercress, ●Chinese Cabbage, ●Cabbage, ●Cauliflower, ●Broccoli, ●Choysum, ●Pakchoi, ●Kai Lan, ●Brussel Sprouts; (**b**) score plot of PCA analysis based on volatile compounds; (**c**) loading plot of PCA analysis based on non-volatile compounds; (**d**) loading plot of PCA analysis based on volatile compounds. The specific names of metabolites are listed in Supplementary Materials Figure S1.

The traditional classification of plants relies more on the morphological taxonomy. Recently, secondary metabolites-based species classification has emerged as an effective chemotaxonomic tool, providing detailed biochemical information on the similarities and differences among plant species [29]. Extensive information of metabolites among the different species of Brassicaceae has been obtained in the discovery set, and the linkage between the morphological taxonomy and the chemotaxonomy was explored. Figure 2 illustrates principal component analysis (PCA) score plots of the 12 vegetables studied based on the non-volatile compounds (Figure 2a) and the volatile compounds (Figure 2b). Although these data are not directly comparable due to different analysis methods used, both datasets show separation of samples from different vegetables into 12 clusters and closer grouping of samples from vegetables of the same genera, with watercress and radishes clearly separate from the other vegetables, and these 12 clusters could further fall into 4 groups according to their morphological classification, as shown in Figure 2a,b. The loading plots of PCA as shown in Figure 2c,d facilitated the visualization of the crucial metabolites for differentiating these Brassicaceae genera. These findings suggest that glucosinolates and their volatile breakdown products could be potential biomarkers for chemotaxonomy, which aligns with classical morphological taxonomy [30].

### 2.3. The Pathways of Glucosinolate Metabolism: From Amino Acids to Glucosinolates and Isothiocyanates

**Figure 3:**
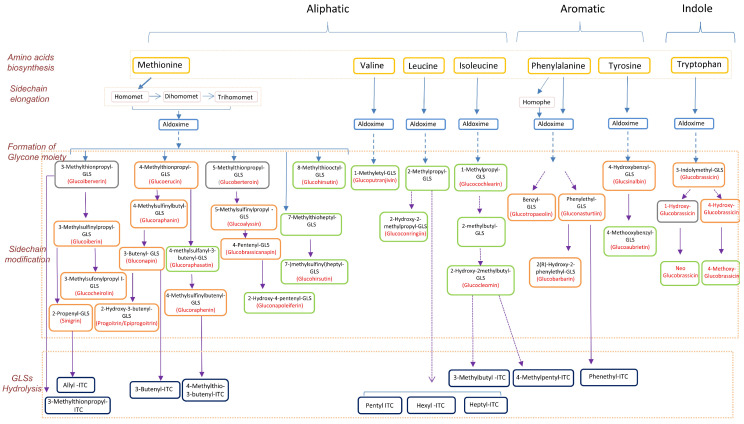
Chart of pathways involving the relevant amino acids, glucosinolates, and isothiocyanates in the 12 vegetables studied. The solid arrow represents the direct step from the upstream metabolites to downstream metabolites, and the dashed arrow represents multiple steps from the upstream to downstream metabolites. Glucosinolates in orange box are quantified with pure standards, glucosinolates in green box are identified by comparison with MS/MS features in the database METLIN (http://metlin.scripps.edu/), and glucosinolates in grey box are not detected in this study. GLS: glucosinolate, ITC: isothiocyanate.

It has been estimated that over 130 glucosinolates are present across plants, and the composition and content varies among families and species [31]. This variation has created a complex picture of glucosinolate biosynthesis that is not easily captured from the literature, which involves a large number of studies published on different aspects of glucosinolate metabolism across genera. In order to have a biologically meaningful understanding of their metabolism, we summarized the available published information together with our present findings into a pathway map depicting the relationships among amino acids, glucosinolates, and isothiocyanates. For practicality and ease of presentation, the map (Figure 3) was limited to those metabolites that were detected and are relevant to the 12 vegetables selected for the present study.

Two databases were used for the pathway map presented in Figure 3, KEGG [32,33,34] and MetaCyc [35]. Additional details were collected from other relevant literature on glucosinolate metabolism [36,37,38,39,40,41,42,43,44,45,46,47].

### 2.4. Three Examples of Distinguished Metabolite Profiles across Specific Pathways

**Figure 4:**
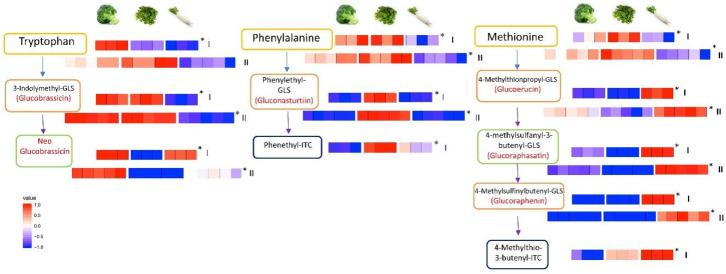
Representative pathway behavior in three vegetables. A heat map of abundances of metabolites detected involved in the pathways from amino acids to glucosinolates and isothiocyanates in three different vegetables which grow in different environments (aboveground broccoli, damp soil or submerged watercress and belowground radish). The data was collected using an untargeted approach and the presented heat maps depict the discovery set of three samples and validation data consisting of 5 samples. The isothiocyanates could not all be included in the validation dataset. The intensities of metabolites in the heat map were expressed as relative levels in broccoli, radish and watercress. Asterisks denote statistical significance by Student’s *t*-test (*p* < 0.05). ITC: isothiocyanate.

To highlight how such large datasets can be used to better understand glucosinolate metabolism in Brassicaceae, we further studied three vegetables from different growing environments to illustrate the differences in metabolic profiles across glucosinolate pathways. Figure 4 shows the three different metabolic pathways of three Brassica plants investigated that are selected from the heat map presented in Supplementary Materials Figure S3. As can be seen, the glucosinolate metabolite profiles differed significantly in the pathways among broccoli that grows above ground, watercress that grows in damp soil, and radish that grows below ground.

When looking at the metabolic profiles across the biosynthetic pathway for the glucosinolate glucobrassicin and its breakdown product neoglucobrassicin, both metabolites are found at higher abundances in broccoli than in watercress or radish. In watercress, a higher abundance of metabolites is detected across the gluconasturtiin pathway and in radish across the glucoerucin and glucoraphasatin pathway. These observations suggest that the pathway from tryptophan to glucobrassicin and then to neoglucobrassicin, is most active in broccoli, whereas the pathway from phenylalanine to gluconasturtiin and phenethyl isothiocyanate appears to be most active in watercress. On the other hand, the pathway from methionine to glucoraphenin and 4-methylthio-3-butenyl-isothiocyanate is most active in radish, among the three species.

## 3. Discussion

In this study, we systematically examined the variations in glucosinolates, their precursors, and their breakdown products among 12 commonly consumed vegetables of the Brassicaceae family using an integrated metabolomics approach.

The results from our principal component analysis (PCA) analysis of the 12 vegetables studied (Figure 2) agreed well with the results from our previous studies [30,48]. In the present study, we are able to distinguish the same 12 vegetables using PCA, based on the profiles of volatile compounds [48] and glucosinolates [30]. For volatile organic compounds, 1490 features were subjected to PCA after cleaning the data of features present in blanks and with too low detection frequencies, with the vegetable samples separated into 12 clusters that correspond to each genus. Further study on glucosinolate profile using PCA of 36 samples also resulted in clear separation into 12 clusters, which can be further clustered into four groups that show close similarity to morphological taxonomical classification.

In order to gain a better understanding of the glucosinolate and relevant synthetic pathways, we constructed a comprehensive map by integrating our data with data from previous studies (Figure 3). The levels of precursor glucosinolates correlate well with levels of their downstream breakdown isothiocyanate products. Moreover, the types and abundances of glucosinolates differ significantly among different genera. This is in reminiscence to previous reports that the glucosinolate profiles of Brassicaceae vegetables could be useful for their taxonomical classification, sometimes even at the level of different cultivars [30,49,50].

Our findings on the three vegetables from different growing environments (Figure 4) reveal that the synthesis of different secondary metabolites in the plant could be either dictated by the growing environment or vice versa, i.e., the plant need to have secondary metabolites to adapt to the growing environment for defense against insects, microorganisms, and nematodes. Further studies are needed to examine how these secondary metabolites react to their potential pests or in different growing environments. Although amino acids are usually considered as glucosinolates’ precursors, we did not observe association between glucosinolates and amino acids (Figure 1).

The correlations in metabolite abundance have shed light on certain functions of a biosynthetic pathway or relationship between precursors and their breakdown products. For example, one of the few amino acids that correlate with alanine is leucine (Figure 1), which can function as alanine’s precursor. However, detecting correlations is by no means necessary for such relationships to exist. At any given time, metabolites may be converted to others, or their synthesis may be regulated by different pathways. Understanding the activity of pathways therefore often requires comprehensive information. For example, Wang and colleagues described the preferential formation of isothiocyanates over nitriles and epithionitriles as species specific, with isothiocyanates being the preferential product in radish (*R. sativus*), but not in *B. rapa* and *B. oleracea*. This is reported to be due to silencing of the epithiospecifier gene, as the putative enzyme it encodes drives the reaction towards the other reaction products [18]. In addition, the authors found genetic differences that help explain the lack of long chain aliphatic glucosinolates and identified genetic factors, which led to tissue specific differences in concentrations of glucoraphasatin and glucoraphanin among the roots, leaves, and seeds.

Nevertheless, our approach is not without limitations. First, the 12 Brassicaceae vegetables studied were obtained from the local market, but were not grown under controlled cultivation conditions. As a result, information on their genotype, growing conditions, and developmental stage at harvest was not available. Thus, it might be hard for others to perfectly reproduce the data obtained in this study. Second, the metabolism of glucosinolates and other plant secondary metabolites could be influenced by both abiotic stress (light, temperature, salt, etc.) [15,22,29] and biotic stress (insect, fugi, bacteria) [13,23,44] during growth. Although it was a surprise that our data showed minimal variations in metabolite profiles among different batches, we cannot exclude the possibility that the content of glucosinolates and their hydrolysis products among different species could have been influenced by environmental factors such as light, water, temperature, insects, etc. Lastly, developmental stage at harvest, packaging, and storage conditions and duration may affect the metabolite profiles as well [23,51,52,53,54].

In fact, self-cultivated vegetables have been recently used in the analysis of glucosinolates and other metabolites in vegetables [18,55,56,57,58], and this approach could prove valuable in the study of glucosinolate metabolism in vegetables and other plants. Related field studies in which growing conditions are well controlled could also be carried out in the future to validate our findings or explore metabolism in Brassicaceae vegetables.

## 4. Materials and Methods

### 4.1. Plant Material

In this study, we used 12 most commonly consumed Brassicaceae vegetables in the Asian region: *Brassica oleracea var. gemmifera* (Brussels sprouts), *B. rapa subsp. chinensis* (pak choi), *B. rapa subsp. pekinensis* (Chinese cabbage), *B. oleracea var. botrytis* (cauliflower), *B. oleracea var. capitata* (cabbage), *B. rapa var. parachinensis* (choy sum), *B. olearacea* var. italica (broccoli), *B. olearacea var. alboglabra* (kai lan), *Raphanus sativus* (daikon radish), *R. raphanistrum subsp. sativus* (red cherry radish), *Eruca sativa* (rocket salad), and *Nasturtium officinale* (watercress). Plant material was obtained from the local supermarkets on the day they appeared on market. The sources of the vegetables are listed in Table S1. It usually takes 5 days for vegetables from harvest to market under standardized transport and storage conditions. Only the edible part was used for analysis: Leaves (cabbage, Chinese cabbage, Brussels sprouts, pak choi, rocket salad), leaves and stems (choy sum, kai lan), florets (broccoli, cauliflower), roots (radish, cherry radish). For non-volatile metabolites analysis, they were immediately washed, wiped, snap-frozen in liquid nitrogen, and freeze-dried after being transported to our laboratory. The resulting dry powder was immediately stored at −80 °C. For volatile metabolites analysis, fresh vegetables were processed and analyzed daily, immediately after being collected from the local supermarkets. The extraction methods and processing of samples for each different type of analysis were done as previously described [30,48,59]. Three samples from the same supermarket were purchased on different days and prepared for each of the 12 vegetables for both untargeted and targeted analysis. Our previous studies on phenolic compounds and glucosinolates of these same 12 vegetables did not differ much, suggesting that they are likely to be from the same sources cultivating with similar methods. Confirmation data was collected on five of the vegetables for five samples each, including the three used as examples of distinguished metabolite profiles in this study.

### 4.2. Chemicals

Methanol, formic acid, ammonium formate, tryptophan, glutamine, asparagine, amino acid standard mixture (AAS18), and isotopically labeled amino acid mix standard (20 amino acids) were purchased from Sigma-Aldrich (St. Louis, MO, USA). Glucosinolates standards including glucocheirolin, progoitrin, glucoraphenin, epiprogoitrin, glucobrassicanapin, glucoalyssin, glucobrassicin, gluconasturtiin, 4-hydroxyglucobrassicin, and glucobarbarin were purchased from Cfm Oskar Tropitzsch GmbH (Marktredwitz, Germany), and sinigrin, glucoiberin, glucotropaeolin, glucoraphanin, gluconapin, glucoerucin, and glucosinalbin were purchased from ChromaDex (Santa Ana, CA, USA). LC-MS grade Acetonitrile was obtained from Merck. In-house purified distilled water was made with a Milli-Q purification system (Bedford, MA, USA).

### 4.3. Untargeted Metabolomics

#### 4.3.1. Analysis of Non-volatile Metabolites

##### Sample Preparation

100 mg of freeze-dried powder samples were treated with 3 mL of 70% methanol containing 20 μg/mL FMOC-glycine as internal standard for 10 min. The extract was centrifuged at 15,000× *g* for 15 min, and filtered through a 0.22 μm nylon filter. 100 μL and 10 μL of the filtrate were used for LC/MS and GC-MS analysis, respectively. For GC-MS analysis, the 10 μL filtrate was dried under nitrogen, and then derivatized with methoxyamine (50 μg/mL in pyridine), with subsequent trimethylsilylation by MSTFA.

The stability of the system was validated using a pooled quality control (QC) sample, which was prepared by pooling 10 µL from each sample. The QC sample was analyzed at the beginning, the end, and randomly throughout the analytical runs.

##### Instrumental Analysis

LC-MS analysis was performed on an Agilent 1290 UHPLC system (Waldbronn, Germany) coupled to a 6540-quadrupole time-of-flight (Q-ToF) mass detector (Agilent, Santa Clara, CA, USA), which was equipped with an electrospray ionization source. The samples were analyzed in both positive and negative ion modes. The elution was conducted at 35 °C on a Rapid Resolution HT ZORBAX SB-C18 Column (2.1 × 50 mm, 1.8 mm, Agilent, Santa Clara, CA, USA) at a flow rate of 0.4 mL/min, with a gradient of 95% solution A (0.1% formic acid in water)-5% solution B (0.1% formic acid in acetonitrile) to 55% solution A-45% solution B over a period of 9 min, followed by 45–100% solution B over a period of 9 min, and subsequently 100% B for 2 min. The autosampler was cooled at 4 °C, and 5 μL of the extract was injected. The following parameters of the mass spectrometer were used for the LC-MS analysis: Ion spray voltage, 4000 V; heated capillary temperature, 350 °C; drying gas flow, 12.0 L/min; nebulizer, 50 psi; collision energy, 10 V, 20 V, or 40 V.

GC-MS analysis was conducted on an Agilent 7890A Series Gas Chromatograph System coupled to an Agilent 7200 Q-ToF mass detector (GC-Q-TOF/MS) and an Agilent 7683B Series Injector kept at 250 °C. The elution was conducted on a fused silica capillary column HP-5MSI (30 m × 0.25 mm i.d., 0.25 μm film thickness). 1 mL of each sample was injected in the splitless mode for the individual analysis. The following parameters of the mass spectrometer were used for the GC-MS analysis: Helium, 1 mL/min; oven temperature, 70 °C for 1 min, 70 °C to 250 °C at an incremental rate of 10 °C/min, 250 °C to 300 °C at an incremental rate of 25 °C /min, and then held at 300 °C for 6 min; transfer line temperature, 280 °C; electron energy, 70 eV; full scan monitoring, *m/z* 50 to *m/z* 550; ion source temperature, 230 °C; quadrupole temperature, 150 °C.

#### 4.3.2. Analysis of Volatile Metabolites

Headspace solid phase microextraction (HS-SPME) combined with GC-MS was employed to analyze the volatile metabolites of the vegetable samples. The SPME fiber (50/30 DVB/CAR/PDMS, Agilent) was conditioned at 270 °C for 1 h before use according to the manufacturer’s instructions. The SPME fiber was exposed for 30 min into the headspace of a vessel containing the vegetable sample immediately after being blended. To avoid potential contamination and capture freshly emitted VOCs, the procedures of SPME fiber preconditioning and sample preparation were optimized to assure that SPME fiber pre-conditioning was performed right before sample blending. Following extraction, the SPME fiber was immediately introduced into the GC injector for 30 s at 230 °C in splitless mode for sample injection.

The same GC-Q-TOF/MS instrument was used for the VOCs analyses, with the elution conducted on a DB-5MS column (30 m × 0.25 mm × 0.25 µm, Agilent, Santa Clara, CA, USA) and the following MS conditions: Helium, 1 mL/min; oven temperature, 40 °C for 2 min, 40 °C to 185 °C at an incremental rate of 5 °C /min, 185 °C to 300 °C at an incremental rate of 30 °C/min, and then held at 300 °C for 2 min; GC total run time, 36.8 min; starting time of data acquisition, 0.5 min; ion source temperature, 230 °C; MS mode, full scan; mass range, *m/z* 35 to *m/z* 500; mass calibration was conducted once every 5 samples to maintain high mass accuracy.

#### 4.3.3. Data Analysis and Metabolites Identification

The data analysis protocol from the literature was employed with minor modifications [60,61]. For non-volatile metabolites identification, the spectral data in each dataset were exported as mzData files and pretreated in open-source software MZmine 2 for peak detection, peak alignment, and peak area normalization.

For volatile metabolites analysis, the GC-MS data were exported as mzData files in Agilent MassHunter Qualitative Analysis software and subsequently uploaded to XCMS online (https://xcmsonline.scripps.edu) for feature extraction and alignment. Normalization of the aligned features was performed by using the fresh weight of the vegetable samples.

After normalization, the aligned features were screened, with only the features in a vegetable replicate showing 100% detection frequency (DF) and low relative standard deviation (RSD < 30%) in peak abundance kept. The features with a p value smaller than 0.05 in Kruskal-Wallis tests were considered statistically significant feature, which were further imported in SIMCA-P 13.0 (Umetrics, Malmö, Sweden) for principle component analysis (PCA) followed by logarithmic transformation. The missing values of the features (i.e., peak area) were replaced by half minimum values.

In LC-MS analysis, the metabolites were identified based on accurate mass, MS-MS spectra, and retention order by matching features in METLIN database (http://metlin.scripps.edu/) with mass errors < 5 ppm. NIST 11 mass spectral library was used to identify metabolites based on retention index and mass spectral similarity (>80%) match. The accurate *m/z* values, RT, and fragmentation patterns are described in supplementary materials (Supplementary Materials Tables S1 and S2).

Student’s *t*-test was performed for comparisons of metabolites among broccoli, watercress, and radish. Pearson correlation analysis was performed to assess correlation between the isothiocyanates (ITCs) and glucosinolates.

### 4.4. Targeted Analysis

#### 4.4.1. Targeted Analysis of Amino Acids

##### Sample Preparation

Approximately 3–4 mg of dried vegetable powders was homogenized in a mixture containing 700 µL of cold methanol and 100 µL of diluted isotopically labeled amino acids internal standard (400 times dilution) in a TissueLyser LT (QIAGEN) (Germantown, MD, USA) for 10 min at 25 Hz. The homogenized mixture was sonicated for 10 min in ice water and then centrifuged at 5 °C at 14,000 rpm for 20 min, with the supernatant subsequently filtered through a Thermo Scientific™ (Waltham, MA, USA)national 750 µL microcentrifugal filter (PTFE membrane, 0.2 µm pore size, non-sterile). 100 μL of the filtrate was dried under 99.9% nitrogen and subsequently reconstituted in 500 μL of acetonitrile/water (75:25, *v/v*) prior to amino acids analysis. Ten quality controls (QCs) of pooled methanol extracts of vegetables were performed along with the samples.

##### Quantification of Amino Acids

The quantitative analyses of amino acids were performed following a previously published protocol [59] on an Agilent 1200 HPLC system coupled to an Agilent (Santa Clara, CA, USA) 6410 Triple Quadrupole (QQQ) mass spectrometer equipped with an electrospray ionization source. Mass spectrum was acquired in multiple reaction monitoring (MRM) mode with a capillary voltage of 3500 V in positive mode, a gas temperature of 350 °C, a gas flow of 12 L/min, and a nebulizer nitrogen gas flow rate of 30 psi. The values of retention time, MRM transition, fragmentor voltage and collision energy (CE), and method validation information are shown in Supplementary Materials Table S3a,b, respectively.

The elution of the amino acids was conducted on an Acquity UPLC BEH Amide column (2.1 mm × 100 mm, 1.7 µm, Waters, Milford, MA, USA)) using mobile phases C (0.1% formic acid and 10 mM ammonium formate in 30% acetonitrile-70% water) and D (0.1% formic acid and 10 mM ammonium formate in 95% acetonitrile-5% water) at a flow rate of 0.5 mL/min with a gradient of 0–1 min, 100% D; 1–2 min, 100–92% D; 2–10 min, 92–85% D; 10–12 min, 85–60% D; 12–14 min, 60–40% D; 14–15 min, 40–15% D; 15–19 min, 15% D; 19–19.5 min, 15–100% D. A column temperature of 40 °C and an injection volume of 5 μL were used for the analyses.

#### 4.4.2. Targeted Analysis of Glucosinolates

##### Sample Preparation

Fresh vegetables were washed, snap-frozen in liquid nitrogen, and then freeze-dried. The freeze-dried vegetables were ground into fine powder and then immediately stored at −80 °C. one hundred mg of each freeze-dried sample was incubated in 1 mL of 70% methanol containing 150 ng/mL of internal standard (Glucosinalbin, Santa Ana, CA, USA) at 70 °C for 10 min. After chilling in an ice bath, the mixture was centrifuged at 15,000× *g* for 15 min, with the supernatant transferred to a new tube. The extraction procedure was repeated twice, and the three supernatants obtained were combined, evaporated to dryness at 40 °C, reconstituted in 3 mL of 70% acetonitrile (ACN), and filtered through a 0.22 μm nylon filter for analysis.

##### Quantification of Glucosinolates

Quantitative analyses of glucosinolates were performed on an Agilent 1290 UHPLC system (Waldbronn, Germany) coupled to a 6490 QQQ mass detector (Agilent, Santa Clara, CA, USA) equipped with iFunnel Technology and an electrospray ionization source. The elution of glucosinolates was conducted on a Waters ACQUITY UPLC^®^ BEH HILIC column (2.1 × 100 mm, 1.7 μm) at a column temperature of 35 °C using mobile phases C and D at a flow rate of 0.4 mL/min with the following gradient: 0–1 min, 100–100% D; 1–5 min, 100–95% D; 5–8 min, 95–80% D; 8–10 min, 80–15% D. The autosampler was cooled at 4 °C, and 5 μL of the extract was injected. The mass spectrometer was maintained in negative ion mode with the following parameters: Drying gas (N_2_) temperature, 200 °C; drying gas flow, 14 L/min; nebulizer, 30 psi; sheath gas temperature, 400 °C; sheath gas flow, 11 L/min; capillary voltage, 3000 V; nozzle voltage, 800 V. Glucosinolates were quantified (with available standards) or semi-quantified (without available standards) in MRM mode with time segments. Data acquisition and processing were conducted with MassHunter software version B.05.00 (Agilent Technologies, Santa Clara, CA, USA).

The quantitative method was validated for limit of detection (LOD), linearity, accuracy, precision, and recovery. Briefly, each standard was dissolved in 70% acetonitrile and diluted to give a series of standard solutions with gradient concentration to make the calibration curves. All the solutions were stored at −20 °C. Method precision was determined by injecting the same mixed sample solution six times consecutively, both in one day for intraday variation and in three successive days for inter-day variation. The reproducibility of the method was determined by analyzing six independently prepared samples from the same mixed vegetable powder. The recoveries were evaluated by spiking glucosinolate standards in three different amounts (approximately equivalent to 0.8, 1.0, and 1.2 times of the concentration in the matrix) into the mixed vegetable sample in triplicate, and were extracted and quantified as described before [48]. The method validation details are presented in Supplementary Materials Table S4.

## 5. Conclusions

The aim of this study was to investigate the glucosinolate pathways of 12 commonly consumed Brassica using both untargeted and targeted mass spectrometry metabolomics approaches. Our findings using this integrated approach not only confirm some of the earlier findings, but also provide much more comprehensive metabolic profiles of several Brassicaceae than those of Brassicaceae studied individually. Integration of these results with data from the literature uncovers that the glucosinolate metabolism in plants from the same Brassicaceae family is significantly distinct in certain metabolic pathways.

Our results have enhanced our knowledge of the glucosinolate metabolism, starting from the amino acids and all the way to their biological beneficial breakdown products, isothiocyanates, and offer useful pointers for a better understanding of the metabolism in different Brassicaceae. Our findings could be useful in efforts to enhance the levels of such compounds in vegetables through plant breeding or other technologies. Our methods and results can serve as an excellent starting point for more detailed investigations into metabolic pathways and their roles in plants.

## Supplementary Materials

The following are available online at https://www.mdpi.com/2218-1989/10/8/313/s1, Figure S1: Heat map of the abundance of metabolites in 12 Brassicaceae vegetables, Figure S2: Metabolic pathways for the detected isothiocyanates and pearson correlation between isothiocyanates (log10 transformed peak area/g) and glucosinolates (log transformed concentration (ng g^−1^)), Figure S3: Heat map of the abundance of metabolites in 3 Brassicaceae vegetables, Table S1: Quantitative results of the amino acids and the glucosinolates in 5 Brassicaceae vegetables (µg/g dry weight), Table S2: Sources of 12 types vegetables, Table S3: MS and MS/MS information of glucosinolates by LC-MS, Table S4: MS information of amino acids and isothiocyanates by GC-MS, Table S5: Method description and validation for amino acids by LC-QQQ, Table S6: Method validation for glucosinolates by LC-QQQ.
