# Supplementary material for: An Integrated Metabolomics Study of Glucosinolate Metabolism in Different Brassicaceae Genera
Source: Metabolites. 2020 Jul 31;10(8):313. doi: 10.3390/metabo10080313 (PMC7463649; doi:10.3390/metabo10080313)
Supplement: Supplementary file 1 [file metabolites-10-00313-s001.zip › metabolites-840743-supplementary-re/Fig.S2.pdf]

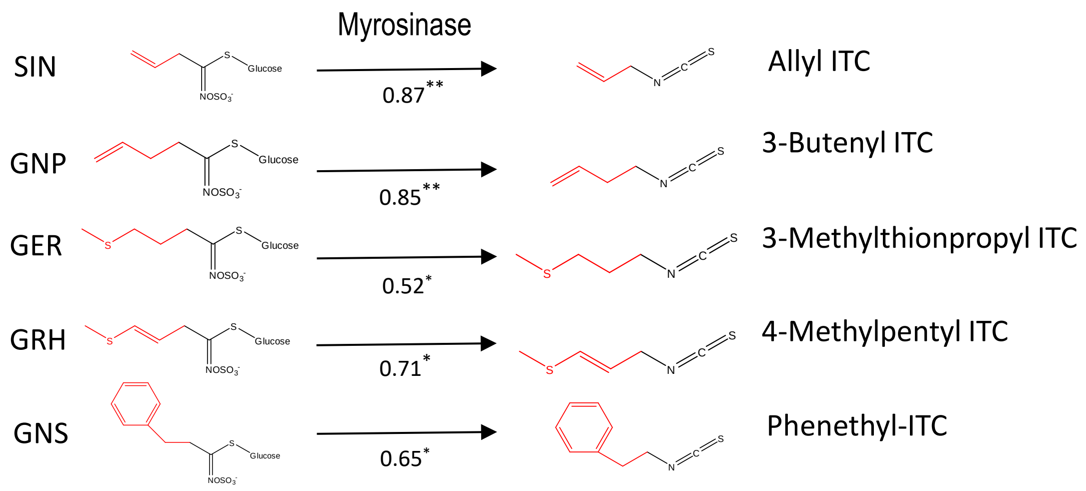

Fig. S2 Relationship between parent glucosinolates and downstream isothiocyanates. Pearson correlation analysis were performed between glucosinolates (log transformed concentration (ng g^{-1}), and isothiocyanates (log10 transformed peak area/g) .The numbers represent correlation coefficient values. *, $p < 0.05$; **, $p < 0.01$. SIN, sinigrin; GNP, gluconapin; GER, glucoerucin; GRH, glucoraphasatin; GNS, gluconasturtiin; ITC, isothiocyanate.
