# Supplementary figures and images for: An Integrated Metabolomics Study of Glucosinolate Metabolism in Different Brassicaceae Genera
Source: Metabolites. 2020 Jul 31;10(8):313. doi: 10.3390/metabo10080313 (PMC7463649; doi:10.3390/metabo10080313)

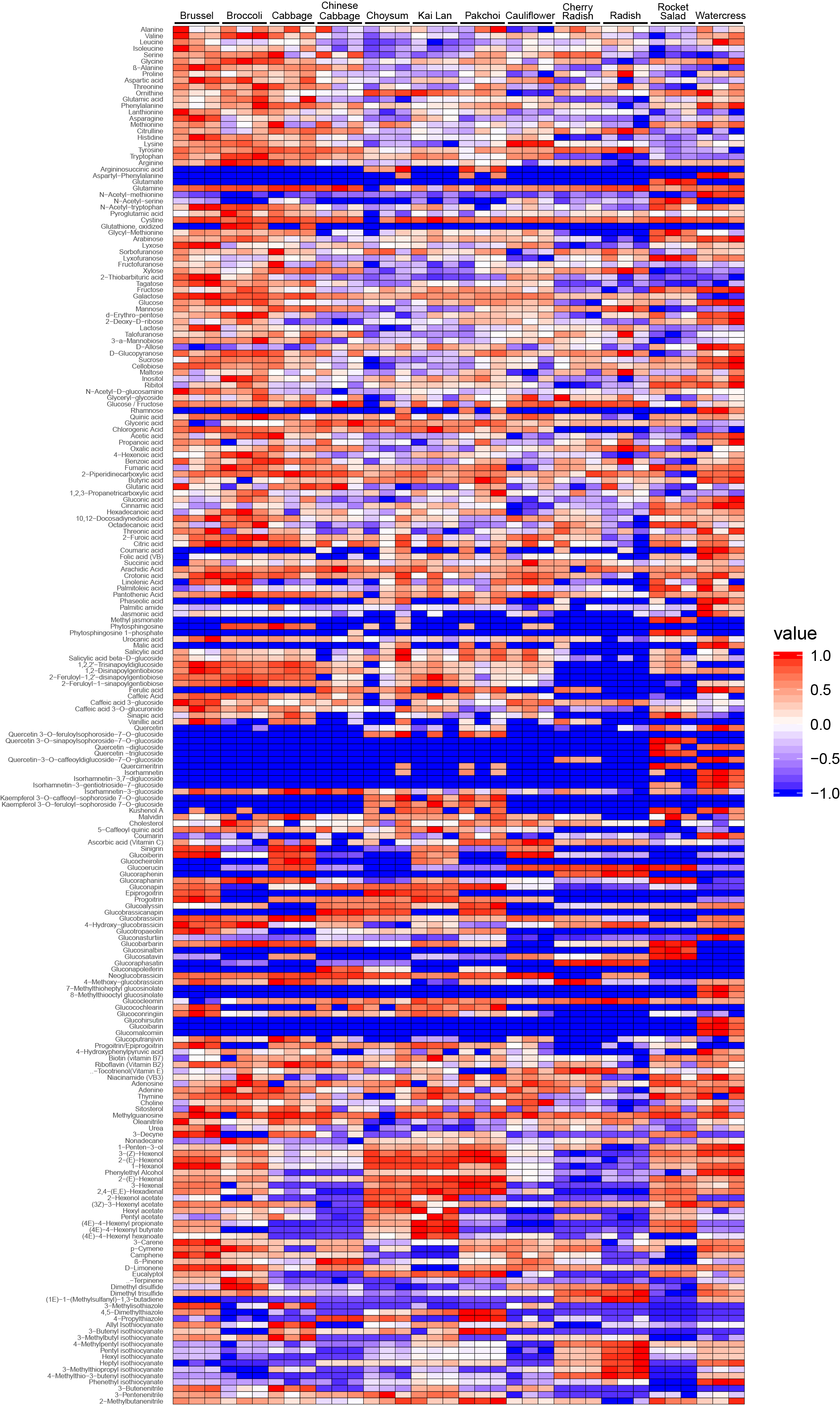

Supplement: Supplementary file 1 [file metabolites-10-00313-s001.zip › metabolites-840743-supplementary-re/Fig.S1.PNG]

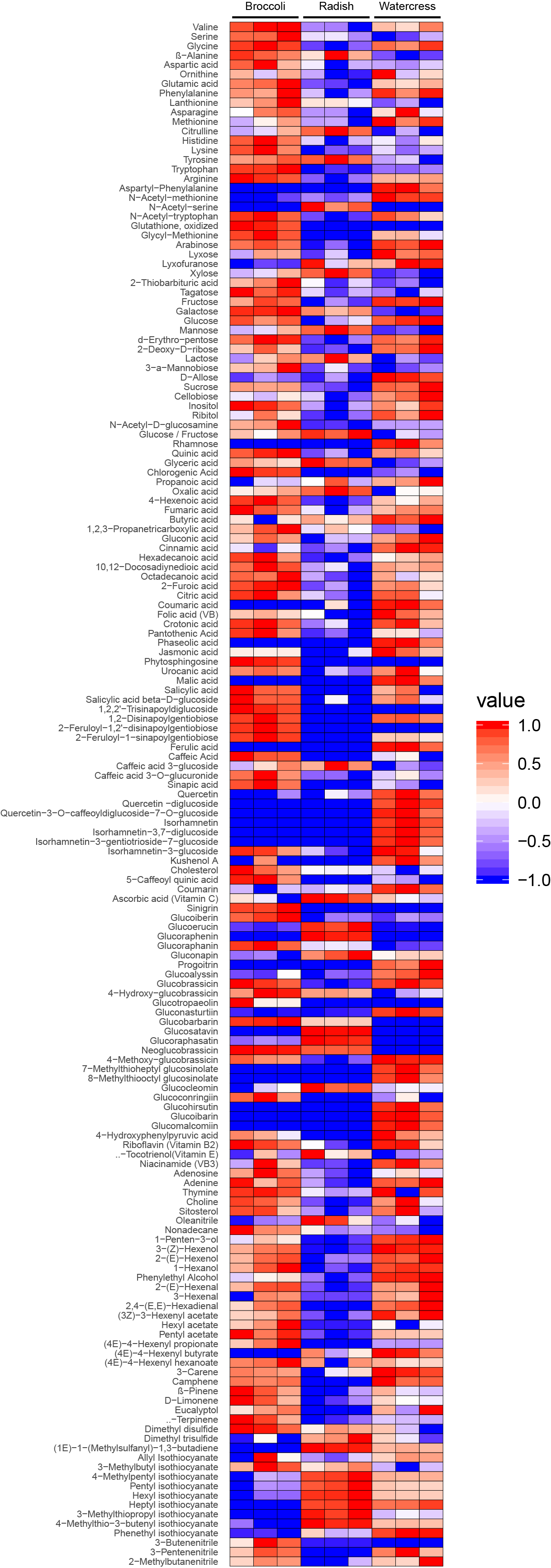

Supplement: Supplementary file 1 [file metabolites-10-00313-s001.zip › metabolites-840743-supplementary-re/Fig.S3.PNG]
